# Are supplemental appraisal/reimbursement processes needed for rare disease treatments? An international comparison of country approaches

**DOI:** 10.1186/s13023-020-01462-0

**Published:** 2020-07-20

**Authors:** Elena Nicod, Amanda Whittal, Michael Drummond, Karen Facey

**Affiliations:** 1 Research Centre on Health and Social Care Management (CERGAS), Bocconi University, Via Roentgen 1, 20136 Milan, Italy; 2 Centre for Health Economics, University of York, York, YO10 5DD UK; 3 Usher Institute, University of Edinburgh, NINE Edinburgh BioQuarter, 9 Little France Road, Edinburgh, EH16 4UX UK

**Keywords:** Rare disease treatment, Orphan medicine, Ultra-orphan medicine, Appraisal, Reimbursement, Access to treatments, Supplemental processes, Health technology assessment

## Abstract

**Background:**

There is increasing recognition that conventional appraisal approaches may be unsuitable for assessing the value rare disease treatments (RDTs). This research examines what supplemental appraisal/reimbursement processes for RDTs are used internationally and how they can be characterised. A qualitative research design was used that included (1) documentation of country appraisal/reimbursement processes for RDTs via questionnaires, desk research and iterative interactions with country experts to produce country vignettes, and (2) a cross-country analysis of these processes to identify and characterise features in supplemental processes for RDTs, and compare them to countries without supplemental processes.

**Results:**

Thirty-two of the 37 invited countries participated in this research. Forty-one percent (13/32) use supplemental processes for RDTs. Their level of integration within standard processes ranged from low to high, characterised by whether they are separate or partially separate from the standard process, adapted or accelerated standard processes, or standard processes that may be applied to RDTs. They are characterised by features implemented throughout the appraisal process. These features are mechanisms that allow application of different standards to assess the value of the medicine, support to the appraisal/decision-making process, overcome the issues of lack of cost-effectiveness, or exempt from part of/the full appraisal/reimbursement process. They increase the likelihood of reimbursement by adjusting and/or foregoing part of the assessment process, or accepting to pay more for the same added benefit as for common conditions. A large proportion of countries with standard processes include one or more of these features (formally or informally) or are discussing potential changes in their systems.

**Conclusions:**

Results suggest revealed preferences to treat RDTs differently than conventional medicines. Some of the challenges around uncertainty and high price remain, but supplemental process features can support decision-making that is more flexible and consistent. Many of these processes are new and countries continue to adjust as they gain experience.

## Background

Health systems are increasingly challenged to fund new and often high-cost medicines in a timely manner [1]. These include medicines to treat rare diseases affecting small patient numbers, which are often severe (life-threatening and/or causing significant disability), genetically acquired and have an onset of symptoms in childhood. In many parts of the world, financial and procedural incentives to facilitate research, development and marketing authorisation have been implemented for rare disease treatments (RDTs) to reduce the high unmet need for disease modifying treatments. In Europe, these incentives are provided by the European Medicines Agency (EMA) through the EU Regulation on Orphan Medicinal Products (OMPs), which was implemented in 2000 targeting medicines treating less than 5 in 10,000 people [2]. These measures are considered to have been successful with the development of 1706 products designated as OMPs, of which 176 have been authorised by the EMA [2].

Decisions relating to reimbursement, pricing and availability of treatments in health systems are often informed by health technology assessment (HTA), which is based on international evidence that is then deliberatively appraised by a committee of experts to determine the added clinical benefit and/or cost-effectiveness of new technologies in a specific health system. The two common challenges in HTA appraisal of RDTs are the uncertainties apparent in the evidence and the high price of the products [3]. The deliberation by the appraisal committee is particularly important for RDTs given the uncertainties resulting from the small and heterogeneous patient populations, lack of information about the natural history of the disease, and the direct and indirect burden of these conditions [4–7]. These uncertainties, combined with the high prices of these medicines (to recoup R&D costs from small patient populations), often lead to cost-effectiveness estimates, such as Cost/Quality Adjusted Life-Year (Cost/QALY), that are much higher than traditional willingness-to-pay (WTP) thresholds [4, 8]. In such circumstances, it may be doubly difficult to justify reimbursement. These aspects have led to increasing recognition that it may be difficult to subject RDTs to conventional HTA processes, particularly in those diseases that require highly specialised care and that are very rare or for so-called “ultra-OMPs” [3, 4, 9–11].

One way to overcome this issue would be to exempt RDTs from HTA altogether, as was the case initially within Germany’s Act on the Reform of the Market for Medicinal Products (AMNOG) system, or pay more for the same added benefit for a patient with a rare disease compared to one with a common condition, as is the case in England’s Highly Specialised Technology (HST) programme [8, 12, 13]. A lower level of scrutiny or a greater willingness to pay could also be seen as acceptable, given the smaller population (and associated budget impact) [11]. However, this has been criticised in the past by some analysts, on the grounds that all health care expenditure deserves thorough scrutiny, or that paying more for health benefit in RDTs could have a high opportunity cost in terms of the services that other patients with common conditions may have to forego [12]. Another way to deal with these issues is to adapt conventional HTA processes to facilitate the appraisal of RDTs. For example, some HTA bodies have stated that the QALY may not capture all elements of value, have adopted a broader decision-making framework and implemented processes to incorporate wider considerations from clinical and patient experts [4, 13]. Other HTA bodies are implementing managed entry agreements (MEAs) to collect outcomes to inform individual treatment decisions, to generate additional evidence for later re-appraisal or to accelerate access to these treatments [14]. Other countries have expedited processes for RDTs that allow patients to get access to these medicines sooner.

As pharmaceutical expenditure is shifting from treatments for more prevalent conditions to medicines that fall within the OMP legislation, the challenges relating to conventional appraisal processes will be faced more frequently [15]. However, the growing number of OMPs are not all for rare, genetic, previously untreatable conditions. Some may be for specific forms of a more prevalent condition, allowing stratified or personalised treatment within a clear patient pathway, whereas others may be very rare conditions where there is no country expertise.

Therefore, the key question is whether supplemental appraisal processes are needed that would better deal with the specificities of RDTs, and if so, what form these should take? This research aimed to address this question by examining how countries are appraising RDTs, with a special focus on those that have implemented supplemental processes specifically for RDTs. This work is part of a Work Package (10) within the EU-funded Horizon 2020 IMPACT-HTA project; it feeds into the overarching objective of the Work Package to develop guidance on novel approaches to appraising medicines to treat rare diseases to support robust and accountable decision-making across Europe on high-cost products.

## Methods

A mixed-methods design was used that included (1) documentation of country appraisal/reimbursement processes for RDTs via questionnaires, desk research and iterative interactions with country experts to produce vignettes for each country, and (2) a qualitative review of these processes to identify and characterise appraisal processes for RDTs and identify the features used in these “supplemental” processes.

The study countries comprised all European Member States and countries from the European Economic Area (Iceland, Liechtenstein, Norway, and Switzerland). Australia, Canada and New Zealand were also included to learn from these jurisdictions with more mature HTA processes. Experts involved in the HTA appraisal/reimbursement processes for RDTs were identified for each country using the authors’ networks or via internet searches. Exchanges were often with just one expert per country, since he/she was often the person with the right competence to provide this information. In a few cases where the responsibility for health care provision is regional (Canada, Spain), several experts for one country were contacted. Experts were invited to participate in this research by email. In case of non-response after numerous follow-up emails, other experts with the appropriate expertise were contacted until appropriate input was received.

A questionnaire with open-ended questions was designed to collect information about country HTA appraisal/reimbursement processes for RDTs, associated challenges, impact and expected policy changes. The questionnaire was reviewed by co-authors (KF, MD) and project partners, and piloted with two institutional partners (National Institute for Health and Clinical Excellence (NICE) in England and the Dental and Pharmaceutical Benefits Board (TLV) in Sweden). Adjustments were made based on the feedback received to ensure the questionnaire was clear and enabled the information of interest to be collected. The questionnaire and accompanying documentation (information sheet and informed consent form) were sent to all country experts by email.

Country vignettes summarising individual country appraisal/reimbursement processes for RDTs were created. If one country had several jurisdictions, these were all included in the same country vignette. Additional desk research was also conducted to confirm and complement some of the information. The country vignette was created and reviewed in an alternating approach by two of the co-authors (AW, EN). Country experts were sent the draft vignettes by email with follow-up questions and opportunity to comment, and for their review and validation on two (or more) occasions, respectively. For some of the countries with RDT supplemental processes, the first validation round was conducted via 1–1 discussions on Skype to allow a more detailed understanding of the features of these supplemental processes to be obtained. The process of creating the vignettes was undertaken from January 2019 until March 2020.

Country definitions of rare and ultra-rare disease treatments were used. In Europe, RDTs are defined by the OMP legislation, but as there is no legislation about ultra-RDTs, this definition differed across countries. In this study, RDTs are defined as all medicines that would fulfil the EMA’s OMP prevalence criteria (medicines to treat less than 5 in 10,000 people), and the term OMP is used when reference is made by the country specifically to those with an orphan designation from the EMA. A process was defined as supplemental if it only included rare and/or ultra-rare disease treatments and related to *routine* use for a defined patient population within a health service. Our analysis did not include for example, named patient programs in which reimbursement is sought for individuals. Similarly, other special processes that are not specifically for RDTs, but that might work to their advantage, were discussed in the section about standard processes if they had been noted by country experts.

The analysis focused on characterising these supplemental processes in terms of (1) level of integration within standard appraisal/reimbursement processes, and (2) their features (unique and/or different) to standard processes. These features were grouped according to their occurrence in the HTA process: (a) categories of *evidence* required, (b) *assessment* of the evidence (including evidentiary standards), (c) *appraisal or deliberative process for decision-making*, and (d) recommendations for *pricing, reimbursement of use of the RDT*. Countries without supplemental processes were then examined to determine whether there were other features within their processes that could be applied to support appraisal of RDTs.

## Results

**Fig. 1:**
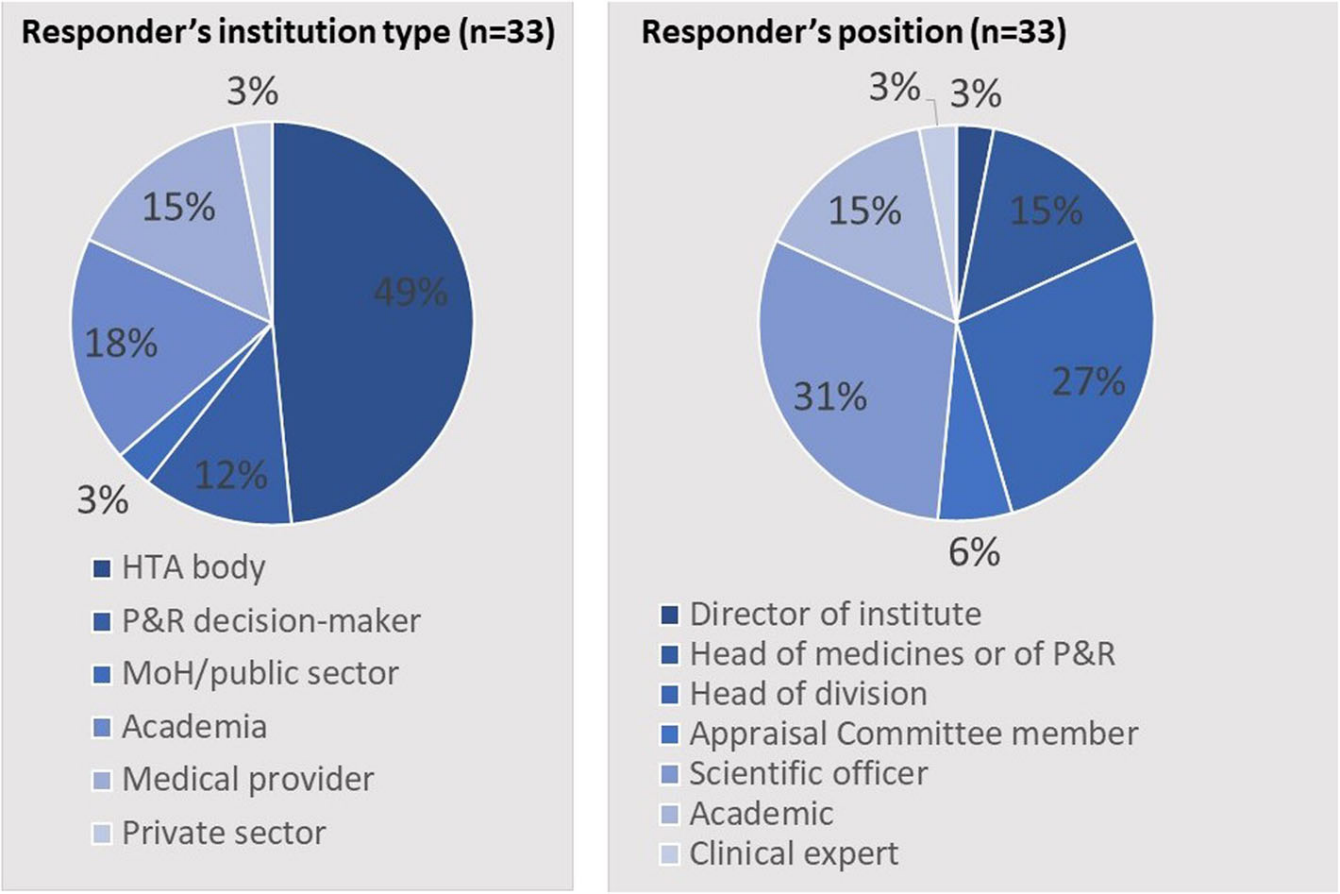
Responder characteristics (*n* = 33)

Thirty-two of 37 countries (86%) were included in the study. Vignettes for each of these were created [16]. The five non-respondent countries excluded from the study were: Australia, Croatia, Cyprus, Luxembourg, and Wales. Responses were provided by 33 country experts (two experts for the different jurisdictions in Canada) (Fig. 1). Eighty percent of experts work within, or close to, HTA/reimbursement processes in the public sector, with the remaining being from academia, health care providers or private sector. Their positions are directorship-level involved in the pricing and reimbursement processes (45%), HTA scientific experts (30%), appraisal committee members (6%), academics or clinical experts (18%), or consultants (3%).

The next section provides an overview of the countries with supplemental processes, explores their key features according to the typology, and examines countries without RDT supplemental processes.

### Supplemental processes for rare disease treatments

**Fig. 2:**
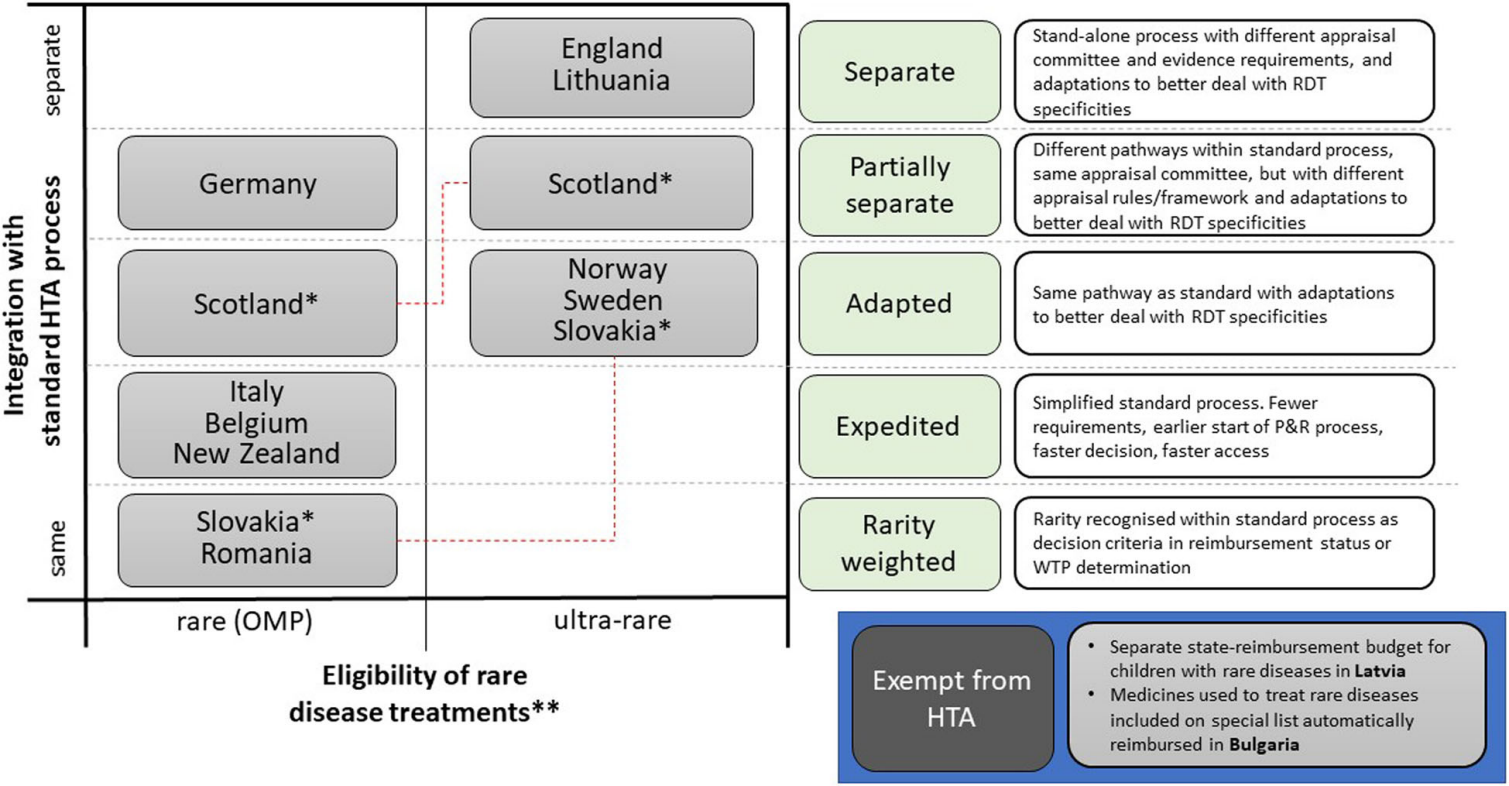
Classification of supplemental processes by level of integration and applicability to rare versus ultra-rare. This figure provides an overview of the study countries that have supplemental processes for the routine use of rare and/or ultra-rare disease treatments in a defined patient population within a health service. * Scotland and Slovakia have two different supplemental pathways for rare and ultra-rare disease treatments respectively, which are differentiated here. ** Rare disease treatment with orphan designation from European Medicines Agency (“Orphan Medicinal Product”, OMP); ultra-rare disease treatment defined by individual country definitions, often alongside other criteria. RDT: rare disease treatment; OMP: orphan medicinal product; P&R: pricing and reimbursement process

Forty one percent (13/32) of countries include supplemental processes specifically targeting rare and/or ultra-RDTs, with two of these countries (Scotland, Slovakia) each having two distinct supplemental processes for rare and ultra-RDTs, respectively (Fig. 2). Integration levels of these supplemental processes within standard processes were categorised as low, medium and high. Processes with low integration are either completely (2/15) or partially separate (2/15) from the standard process. The main distinction between separate supplemental and standard processes are the different evidence submission requirements and appraisal committees. This is the case for the HST programme in England and the ultra-OMP pathway in Lithuania, both targeting ultra-RDTs. They also include different features intended to better adapt to the specificities of RDTs. Both recognise the challenges for evidence generation and tend to be more lenient with its interpretation. The HST programme in England has an explicit decision-making framework that allows for broader consideration of treatment value (nature of the condition, clinical effectiveness, value for money, impact beyond direct health benefits) and greater WTP, whereas the ultra-OMP pathway in Lithuania has different appraisal rules (therapeutic value not graded), a special reimbursement list with no waiting list, and special pricing rules.

Partially integrated supplemental processes include the ultra-OMP pathway in Scotland (where the wider decision-making framework implemented in 2016 was changed to an ultra-OMP pathway in 2019) and the OMP pathway in Germany. In Scotland, once a product is designated as an ultra-RDT by the Scottish Medicines Consortium (SMC), it is submitted for initial assessment to SMC using a special submission form and it is then made available in Scotland for 3 years with a data collection plan and re-assessment after 3 years. In Germany, the assessment and appraisal are conducted by the Federal Joint Committee (G-BA) instead of the independent Institute for Quality and Efficiency in Health Care (IQWiG). RDTs are also subject to more simplified evidentiary requirements (e.g. no need for comparative data), and their additional benefit is considered automatically proven. There is also an option for conditional approval.

Countries were defined as having *adapted* processes for RDTs when they include adjustments to their standard process that allow better management of the challenges with RDTs. This is the case for Norway, Sweden and Slovakia, in which the adapted process targets ultra-RDTs, and in Scotland for OMPs. The main process features common to all the countries relate to a greater understanding of the challenges to produce high quality evidence for RDTs, and more favourable reimbursement through a higher WTP. Slovakia also includes an exemption from presenting an economic evaluation. In Scotland, being an OMP has been seen as a “modifier” to the appraisal process for many years, providing more flexibility in the decision-making process [17]. Recently, there is also the possibility to hold a patient and clinician engagement (PACE) meeting for OMPs [18].

Five of the 13 countries with supplemental processes for RDTs have a high level of integration with standard processes. These are either *expedited* processes or processes where *rarity is weighted*. The three countries with expedited processes allow for an earlier start of the assessment process (and subsequently earlier access for patients to these treatments). The other two countries have points systems to determine reimbursement status (e.g. Romania) and WTP (e.g. Slovakia), where OMPs get extra points.

Two of the 13 countries with supplemental processes use alternative routes to reimburse subgroups of RDTs, through *HTA exemptions*. These include the separate state-reimbursement budget for children living with rare diseases in Latvia and a list of rare diseases for which medicines are automatically reimbursed in Bulgaria.

### Distinction of rare versus ultra-rare disease treatments within supplemental processes

All processes with a medium to low level of integration within standard processes target ultra-RDTs, with the exception of the orphan medicines pathway in Germany and the PACE process and SMC modifiers in Scotland intended for OMPs (Table 1). The ultra-RDT definitions are defined using prevalence criteria ranging from less than 1 in 50,000 (Scotland, Slovakia) to less than 1 in 200,000 patients (Lithuania, Bulgaria), or are not defined (Sweden, likely to be less than 1 or 2 in 200,000 patients). Additionally, most countries limit these special processes to ultra-RDTs with specific characteristics, such as treating a severe condition with high unmet need, to potentially effective and/or high cost treatments, requiring highly specialised management. Regarding the more integrated supplemental processes, eligible medicines are those with an OMP designation.

**Table 1:** Country definitions for rare and ultra-rare disease treatments, and their use as eligibility criteria within supplemental processes for rare disease treatments

PROCESS TYPE	COUNTRY	PROCESS DESCRIPTION	ELIGIBILITY	DEFINITION	
				Rare disease	Ultra-rare disease
Separate	**England** Highly Specialised Technology Programme (HST)	Main differences with standard process: willingness to pay threshold, specialised appraisal committee, more holistic perspective of value, managed access agreements possible	High cost technologies for ultra-rare conditions - see HST prioritisation criteria	–	No prevalence criteria, based on HST eligibility criteria
	**Lithuania** Ultra-OMP pathway	Very rare disease committee: special appraisal committee decides on inclusion in special list. Main differences with standard process: therapeutic value not graded, no waiting list in case of positive decision, special pricing rules. Decision can be individual-case (yearly revised fixed budget) or generalised-case approach (general budget)	(1) ultra-rare, (2) life-threatening or significant disability, (3) subject to effective aetiology or pathogenic treatment, (4) effective treatment (increases survival or reduces disability)	–	< 1:200,000
Partially separate	**Scotland** "Ultra-OMP pathway	Assessment based on ultra-OMP decision-making criteria, routine use for 3 years after which re-assessment. Option for PACE process. Disease-specific experts describe treatment benefit not captured within original assessment	URDT: (1) ultra-rare, (2) chronic and severely disabling condition, (3) highly specialised management PACE: OMPs (and end of life treatments) not considered cost-effective - after NDC decision (after 3-year monitoring)	–	< 1:50,000
	**Germany**	Different reimbursement status: additional benefit guaranteed, strong negotiation power and reasonable reimbursement. Assessment by G-BA (instead of IQWIG), different evidence requirements	(1) OMP, (2) revenues from statutory health insurance < 50 million/last 12 months	OMP	–
Adapted	**Norway**	Greater willingness to pay	(1) ultra-rare, (2) effective treatment (> 2 QALY gain), (3) severe condition (> 30 QALYs lost)	–	< 1:100,000
	**Slovakia**	Exempt from economic evaluation	Ultra-rare	–	< 1:50,000
	**Sweden**	Greater willingness to pay	(1) ultra-rare, (2) good potential for effective drug, (3) very severe condition	–	no fixed limit ~ < 1–2:100,000
	**Scotland** Standard pathway with PACE and modifiers	PACE: disease-specific experts describe treatment benefit not captured within original assessment. Modifiers: standard assessment for OMPs, but SMC recognises limitation in evidence generation and will accept greater uncertainty in the economic case	PACE: OMPs (end of life treatments) not cost-effective, manufacturer can request a PACE to get additional insights Modifiers: OMPs, life-threatening, substantial increase in quality of life/life expectancy, can reverse the condition, bridges gap to a definitive therapy.	OMP	–
Expedited	**Belgium**	Earlier pricing: after positive CHMP opinion, before marketing authorisation. Exemption from economic model	OMP	OMP	–
	**Italy**	Earlier pricing and reimbursement: after positive CHMP opinion, before marketing authorisation	OMP (and hospital or exceptionally therapeutic and social medicinal products)	OMP	–
	**New Zealand**	Earlier reimbursement: before marketing authorisation	Rare disease (as per country definition)	Cum prevalence < 1:50,000	–
Rarity weighted	**Romania**	Reimbursement status based on points cumulated (unconditional, conditional reimbursement etc.): OMPs get extra points	OMP	OMP	–
	**Slovakia**	Willingness to pay threshold based on points system: OMPs get extra points	OMP	OMP	–
Exempt from HTA	**Bulgaria**	All drugs to treat those rare diseases included in special list of rare diseases are 100% reimbursed	Drugs with indication included on special list of rare diseases	OMP	–
	**Latvia**	Separate state-reimbursement budget for children with rare diseases	OMP for use in children	OMP	–

Legend: *HTA* Health technology assessment, *OMP* orphan medicinal product (refers to drugs with an orphan designation from the European Medicines Agency), *RDT* rare disease treatment, *HST* Highly Specialised Technology programme, *SMC* Scottish Medicines Consortium, *PACE* Patient and Clinical Engagement programme, *G-BA* Federal Joint Committee, *IQWIG* Institute for Quality and Efficiency in Health Care, *CHMP* Committee for Medicinal Products for Human Use of the European Medicines Agency, *NDC* New Drugs Committee, *QALY* Quality of Life Adjusted Life Years

### Key process features for appraisal of rare disease treatments

**Fig. 3:**
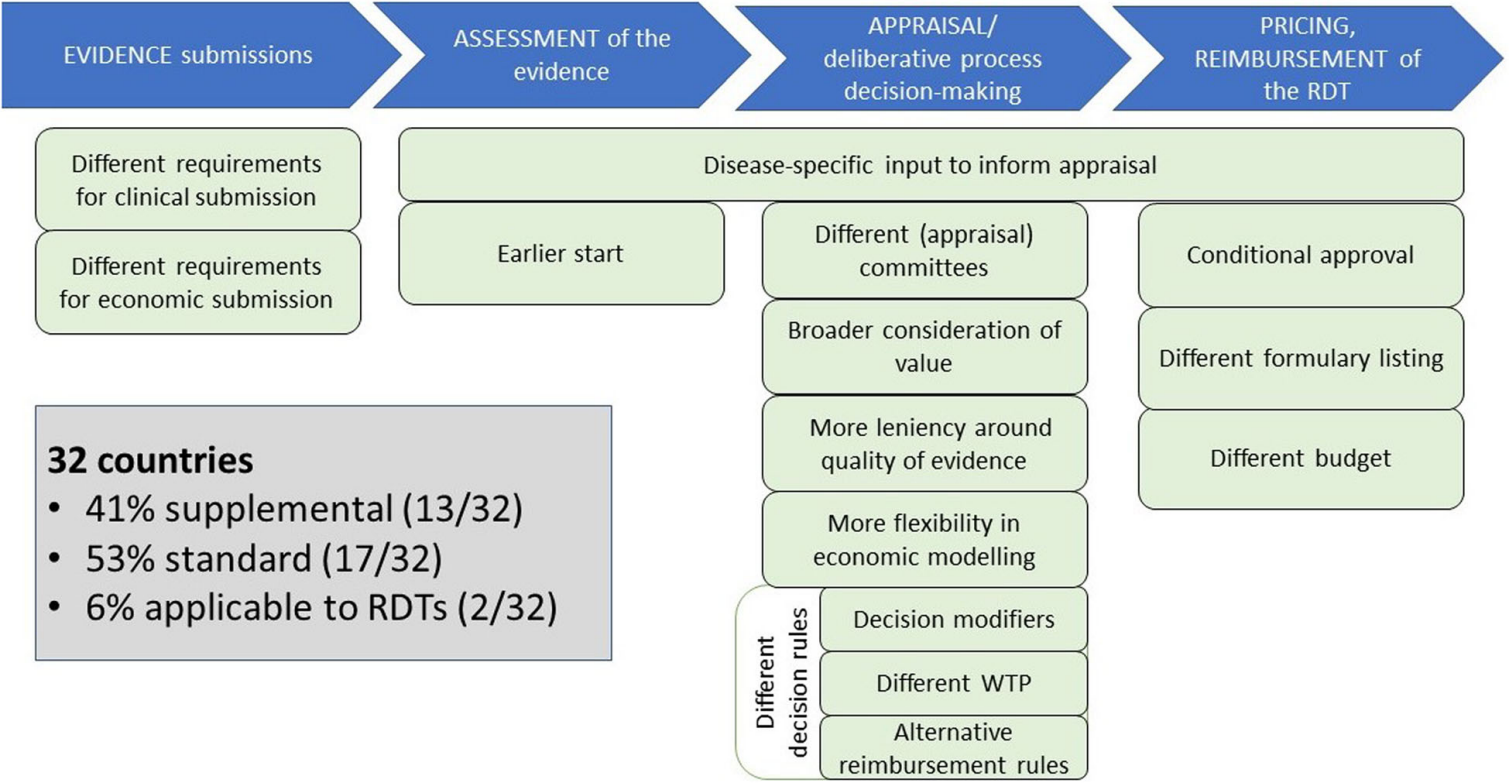
Features included in supplemental processes for rare diseases across the HTA process. Illustrates the (unique and/or different) features of supplemental processes to standard ones. They have been categorised according to their occurrence throughout the HTA process. RDT: rare disease treatment; WTP: willingness-to-pay

The distinctions between supplemental and standard processes have been characterised as features occurring throughout the HTA process, and are discussed in this section (Fig. 3).

#### Evidence submissions

The countries with separate or partially separate processes have different clinical and/or economic evidence submission requirements. This is done through use of different submission forms as seen for the HST in England and ultra-OMP pathways in Lithuania and Scotland, or with the possibility of presenting a simplified version of the standard submission in Germany (exempt from presenting comparative data). In all other countries, evidentiary requirements are the same as for standard processes with the exception of Lithuania, Slovakia and Belgium, which don’t require economic models for ultra-RDTs.

#### Assessment

The inclusion of disease-specific input is being achieved by involving patient and clinical experts in different ways across the HTA process, starting from the assessment phase. First, through the stand-alone PACE process in Scotland, where the SMC assessors discuss the added benefit of the treatment not captured within conventional clinical and cost-effectiveness assessments with those who have experience of the disease and/or treatment being assessed. This meeting can be requested by the manufacturer if a negative opinion is initially given by the assessment committee. A PACE statement is then drafted and becomes part of the evidence submitted to the appraisal committee. Secondly, the process may allow for clinical (and in some cases patient) experts to provide input about the condition, the care pathway in the country and impacts of treatment in standard clinical practice, which supports understanding and interpretation of the evidence and assumptions. Most countries, including countries with standard processes for RDTs, allow this. Third, some countries have established committees with rare disease experts that support decision-making around pricing, reimbursement and use of the medicine. This is the case in Bulgaria with their rare disease expert committee that decides on inclusion of diseases on the special positive formulary (for which all treatments are reimbursed), and in New Zealand with their clinical advisory committee.

Additionally, some processes may allow for an earlier start in the assessment process to guarantee more timely access to RDTs. This is seen in Belgium, Italy and New Zealand in their expedited processes targeting RDTs, which allow for the assessment process to start before marketing authorisation is granted.

#### Appraisal

The greatest number of features implemented in the supplemental processes relate to the appraisal phase. The main distinction seen within separate supplemental processes for ultra-RDTs is the existence of different appraisal committees, which provides a standing group with rare disease expertise who only assess RDTs. One of the main distinctions seen in England is in membership composition with the inclusion of rare disease clinical specialists (adult and paediatric), ethicists, geneticists, and rare disease expert centre representatives.

Broader consideration of value is another feature adopted in England’s and Scotland’s supplemental processes for ultra-RDTs (within their ultra-OMP decision frameworks), where context and specification of issues specific to rare diseases are considered as evidence. This is done by more detailed consideration about the nature of the condition (including consideration of severity, unmet need and existence of alternative treatments), and accounting for indirect costs and benefits on patients, carers and health system.

The most common feature in supplemental processes relates to allowing more leniency in the quality of the evidence. This is done through less stringent requirements for demonstrating added benefit (e.g. acceptance of non-comparative data), and/or willingness to accept surrogate endpoints or non-randomised controlled trials (RCTs). Generally, this is done on a case-by-case basis, if appropriate justification is provided or if the evidence is considered to be of best possible quality. Greater leniency may also be more acceptable for medicines with a high level of unmet need or those that have a high media profile.

In those countries where cost-effectiveness needs to be proven (e.g. where an economic submission is required), there may be more flexibility in the interpretation of the economic evidence. This is done by accepting natural outcome measures instead of QALYs or cost-consequence analyses, and including sensitivity analyses that reflect wider costs and benefits (Scotland) [19].

Some of the features included in supplemental processes allow for different decision rules. The first relates to different WTP, where a higher ICER than would be admissible in common conditions (Scotland, Norway, Sweden), or a higher adjusted WTP that increases with magnitude of benefit and QALY gained (England) would be accepted, or through a points-system calculation of the WTP threshold where OMPs get extra points (Slovakia). The second relates to alternative reimbursement rules, where the therapeutic benefit of the medicine would be considered proven (Germany), or would not be graded (Lithuania). In Romania, reimbursement status is based on a points system, where OMPs get extra default points. Third, the process features also include decision modifiers in the appraisal framework, which impact deliberation about WTP or reimbursement status. While most processes, including the standard ones, are mainly interested in severity, unmet need and existence of treatment alternatives, some of the processes explicitly account for rarity or other criteria that may favour appraisal of RDTs (e.g. children, equality or the innovative nature of treatment [20]). Rarity is accounted for in Scotland as part of its decision modifiers. Other criteria that may influence the decisions are equality (England, New Zealand), children (Germany), ethical considerations (Bulgaria, Latvia), or innovative nature of treatment (England, Italy).

#### Pricing and reimbursement

Many countries include different forms of conditional approval agreements or MEAs, aiming to collect additional data to facilitate later re-assessment of added benefit or cost-effectiveness. This is the case for England’s HST programme, Scotland’s ultra-OMP pathway, Germany, Norway, Slovakia, Italy and Belgium. A few countries also include alternative routes to pricing and reimbursement for a group of RDTs, where they would be exempt from HTA as a whole. This is the case in Bulgaria, where all medicines used to treat conditions included on a special list of rare diseases would be automatically reimbursed, and in Latvia, with their separate state-budget for children with rare diseases.

### Impact and proposed changes

Country experts were asked to state the impacts of their supplemental processes for RDTs. Increased acceptance rate was the most common response, resulting in the reimbursement of medicines that otherwise would not be reimbursed. The assumption of proven added benefit was considered to ensure a stronger negotiating position for the manufacturer and more flexibility in pricing. Some countries stated that these processes are more adapted to dealing with specificities in appraisal of RDTs. These processes allow special approaches to be taken for reimbursement of RDTs with clinical and/or budgetary issues in a consistent way, rather than on a case-by-case basis, supporting fairness in the decision-making system.

Changes in four of the 13 countries with supplemental processes are also being discussed, including refinement of processes (Sweden), potential legislative changes (Romania), implementation of a separate budget for adult rare disease patients (Latvia) and establishment of a therapy monitoring process (Bulgaria).

### What are countries with standard processes doing?

In total, 19 countries (out of 32) do not have supplemental processes targeting RDTs, but may have features in their standard processes that might work to the advantage of RDTs. Ten (out of 19) adopt one or more of the features identified, but these are not specific to RDTs. Nine countries (of the 10 countries adopting one or more of the features) have a process that allows more leniency around quality of evidence (Austria, Canada, France, Estonia, Hungary, Poland, Portugal, Czech Republic, and Netherlands). Two (out of 10) countries have different clinical and economic submission requirements (Estonia, Czech Republic). Two (out of 10) countries have a process that allows for more flexibility in the use of the economic model in decision-making (Estonia, Slovenia). For example, Estonia has a process for accepting different WTP. Six (out of 10) countries can implement conditional approval schemes (Austria, Canada, Ireland, Czech Republic, Netherlands). Two (out of 10) have separate budgets (Italy has a separate fund for innovative and cancer medicines and Austria has specific funds for political solutions). Ireland has a Rare Diseases Technology Review Committee enabling clinicians and other stakeholders to provide input in the post-HTA phase, and review MEA proposals (was not included as a country with a supplemental appraisal process, since this applies to post-HTA/appraisal). Some of these 10 countries and two without process adaptations (Finland and Greece) have appraisal criteria likely to favour RDTs. These relate to rarity (Netherlands), equality (Netherlands), children (Netherlands), ethical considerations (Netherlands, Slovenia, Finland), or innovative nature of treatment (Greece).

Some countries include additional processes that are not specifically for RDTs, but might work to their advantage. In the Czech Republic, highly innovative medicines are eligible for conditional reimbursement, and do not need to prove cost-effectiveness. Switzerland automatically grants reimbursement for diseases included in the national list of birth defects and congenital disorders [21]. In France, their expedited processes target medicines with high unmet need.

Eleven of the 19 countries without supplemental processes are engaged in ongoing discussions about implementing changes in their processes for RDTs. Discussions revolve around general process changes in the standard pathway, or process changes for orphan medicines, including implementing exemptions for economic models or including bespoke patient involvement processes.

## Discussion

This research contributes to a better understanding of how countries’ reimbursement/appraisal processes are dealing with RDTs. The distinctive contribution is in the primary nature of the data collected from experts closely involved within these processes, which allow the first comprehensive and detailed review of the supplemental processes targeting RDTs in Europe and beyond. This issue has been previously explored through systematic reviews or case studies, however, these earlier findings target a narrow sample of countries (with scarce information on Eastern European countries [6]), and focus on their evaluation criteria [6, 22–24], and/or on governance structures for pricing and reimbursement [24]. This study additionally identified and characterised the process features that facilitate appraisal of RDTs. A range of features impact access to orphan medicines across countries and this paper has not sought to address those, its focus is simply on appraisal/reimbursement systems.

Forty-one percent (13/32) of countries include supplemental processes for RDTs characterised by (add-on or adjusted) process features. This section discusses how these features help deal with uncertainty and price, and, on this basis, whether supplemental processes are needed for RDTs in all appraisal systems. It explains how the features can support application of different standards, resolution of uncertainties, lack of cost effectiveness and expedited decisions.

### Different standards

*The features that are specific to RDTs allow for different standards to demonstrate the value of RDTs****.*** This is reflected in the different submission requirements, standards in assessing the quality of the evidence, value assessment frameworks, and decision rules around determination of value.

First, different submission requirements may enable submission of evidence that would not normally be acceptable or additional information to augment the clinical evidence base. For example, no comparative data are required for OMPs in Germany as their added benefit is assumed to be proven. However, some manufacturers may choose not to use this route as those medicines for which there are no comparative data are likely to be categorised with “non-quantifiable” added benefit, which may render price negotiations more challenging [25, 26].

Secondly, when assessing the quality of evidence, most HTA bodies use standard processes that take no account of the small populations available to study and the limitations caused by the lack of clinical and epidemiological understanding of a rare disease that is needed to develop a robust evidence base [27]. This has been shown in different reviews of HTA methods guides that identified only a few exceptions to standard methods for rare diseases [11, 28]. We identified a few countries that have some adjustment to their assessment processes for RDTs, undertaking special consultations with experts to feed into the appraisal deliberations. However, it is unclear how well targeted these expert engagements are and whether they have the standing in the process to influence the confirmation or rejection of assumptions that drive the understanding of value.

Thirdly, different value assessment frameworks considered within England and Scotland’s ultra-OMP pathways allow for consideration of a wider perspective of treatment value. This may help overcome limitations of QALY-based approaches that may lack sensitivity in assessing value [7, 29], or management of high levels of uncertainty characteristic of rarer and more severe conditions [29, 30]. A structured appraisal framework for RDTs could include wider perspectives, as well as guidance on how to account for these in determining added benefit during decision-making. This is particularly important to clarify for the qualitative considerations of evidence, which includes non-quantified (e.g. where data may be lacking) or non-quantifiable considerations (e.g. where no appropriate measure exists to quantify a criterion), as they tend to be given less weight in appraisal compared to quantitative evidence [11].

Fourthly, the different decision rules around determination of value are another way to forgo part of the HTA process to determine added benefit and grant reimbursement.

### Resolution of uncertainties

*These features can help resolve uncertainties in the interpretation of clinical (and related economic) evidence.* First, some processes allow for disease-specific input to inform the appraisal by gathering additional information not included in the original submission (e.g. PACE meeting in Scotland), by receiving input on clinical issues (e.g. plausibility of clinical assumptions, relevance of outcomes and meaningfulness of improvement [13]), or by having special rare disease committees to support pricing, reimbursement and treatment use decisions. Secondly, specific appraisal committees composed of decision-makers with experience in rare diseases may better understand some of the specificities with RDTs given their experience accumulated across very rare conditions (England and Lithuania). Thirdly, most countries (with and without supplemental processes) recognise the challenges to generate robust evidence for RDTs and the implications this will have for increased uncertainties and so are willing to be more flexible in interpretation of evidence by, for example, assessing if it is the best quality evidence based on the feasibility of conducting RCTs and comparisons. Fourthly, although conditional approvals requiring some form of evidence generation post HTA approval have proved challenging in the past, with improvements in collection of real-world data and sharing of experiences across countries [31], there is renewed interest in their application, particularly for highly innovative RDTs.

### Lack of cost effectiveness

*These features help overcome the issue of lack of cost-effectiveness.* This can be done by accepting different WTP levels or granting exemption from submitting a cost-effectiveness model, which may be a way to recognise the unsuitability of conventional approaches in appraising RDTs. Different WTP for RDTs would consist in accepting higher and/or adjusted thresholds, or in other words, by accepting to pay more for the same level of benefit compared to common conditions. Some countries, however, are still faced with cost effectiveness estimates above the revised WTP threshold. Another approach to dealing with this issue is by granting more flexibility in the use of the economic model, accepting other types of models such as cost-consequence, recognising that it is not always possible to populate economic models, using alternative pricing approaches, or giving less weight to the economic model in appraisal. Some countries do not require an economic model for certain types of medicines (e.g. highest level of added benefit or highly innovative medicines), coupled with other measures to manage risk (e.g. under conditional approval). Generally, an economic model may provide a useful benchmark for some. Indeed, the Institute for Clinical and Economic Review in the US considered removing the requirement for an economic model when reviewing their Value Assessment Framework for ultra-rare conditions, but decided to keep it, due to its usefulness in decision-making, alongside consideration of other relevant information not captured within the model [32]. This brings us to one of the key issues about how these additional elements, often difficult to quantify, may influence decision-making particularly when cost-effectiveness is not demonstrated (even within an “adapted” processes for RDTs).

### Accelerated processes

*These features aim to expedite the HTA/reimbursement decision to guarantee more timely access to patients to RDTs despite the uncertainty in the evidence base.* First, HTA processes can be started before marketing authorisation is granted to enable an HTA decision soon after CHMP decision. However, this can be challenging for a RDT where the submitted indication may be more likely to be amended given paucity of data and implications for the benefit:risk profile. This would then require an alteration in the HTA submission to be in line with the approved indication. Secondly, some countries forgo the HTA process by granting access via separate budgets or rare disease positive lists. However, separate routes, such as ear-marked budgets, heavily rely on political support and risk being revised or discontinued [10], particularly in a time when the proportion of pharmaceutical expenditure is likely to increase for RDTs and other high cost medicines [33].

### Do we need supplemental processes for RDTs?

These results do not outline what an ideal process for RDTs looks like, but identify some of the key features that can be implemented to facilitate this process. Our results indicate revealed preferences to treat RDTs differently in many jurisdictions. Indeed, 78% of the 32 study countries are dealing with RDTs differently: 41% (13/32) of countries have supplemental processes for RDTs, 31% (10/32) of countries with standard processes utilise other special features facilitating appraisal of RDTs, and 6% (2/32) of countries account for appraisal criteria likely to favour RDTs. Of the seven countries remaining, four are planning changes in their systems. The high number of countries with standard processes that are applying some of the features facilitating appraisal of RDTs or that are planning changes in their systems suggests that these countries are adopting similar approaches to supplemental processes, but in a less systematic and more ad hoc manner.

Despite the observed inter-country heterogeneity, the features implemented within supplemental processes aim to manage the specificities of RDTs fairly and consistently. They allow for different standards to demonstrate the value of RDTs, support the interpretation of limited clinical (and related economic) evidence, help overcome the issues of lack of cost-effectiveness, and aim to accelerate the HTA process to guarantee more timely access to RDTs for patients. Their impact increases the likelihood of reimbursement by adjusting and/or forgoing some part of the assessment process, and by being willing to pay more for the same added benefit as for common conditions, which is also likely to lead to accepting high prices. They apply to ultra-RDTs and/or RDTs, and are often applied in combination. Having supplemental processes does not mean that all issues are being dealt with. The challenges around uncertainty and high price remain, but these process features allow for more consistent, adapted and flexible decision-making processes and improved accountability for reasonableness [34]. Many of these processes are new and countries continue to adjust them as they gain more experience in their use.

As regional and pan-European cross-border Payer/HTA collaborations, such as Beneluxa, Finose, Valletta Declaration or Eunethta [35–38] are being implemented to conduct joint value assessments, one of the key questions is whether RDTs should have a common appraisal framework and/or undergo a common appraisal process? A first consideration is the extremely fragmented and complex market in Europe, which makes it difficult to navigate particularly for small/medium-sized companies. Secondly, national evidence generation may be more challenging for rare diseases with a limited patient population. Under such circumstances, a common appraisal framework more adapted to rare disease specificities in terms of the type of evidence to submit and the approaches to deal with uncertainty would be beneficial. Further discussion about wider perspectives of value, or the types of reimbursement decision rules would also be useful, although under the principle of subsidiarity, these items are likely to remain within the competence of EU member states. Additionally, in light of the nature of some of these very rare conditions, which in some cases can affect 10 or fewer patients in a given country, it could seem counter-productive for a company to have to produce a bespoke submission for each individual country. In such circumstances, some form of joint approach to appraisal/reimbursement could be more efficient and less time- and resource-consuming for all parties involved.

The results presented in this paper do not outline what an ideal appraisal framework looks like. It represents a first step to understanding how countries are dealing with RDTs and how they can learn from each other about the different ways to adapt standard processes to better deal with RDTs. This could then be used to shape a common appraisal framework and be useful when implementing cross-border joint assessment collaborations.

## Limitations

A number of challenges arose when compiling the vignettes and analysing the data collected. Responses to questionnaires may have included an insufficient level of detail and/or absence of links or documentation, making it difficult to grasp the full context and complexity of the systems in different countries. In order to minimise the risk of missing relevant information, desk research, feedback from institutional partners, and several rounds of validation with country experts were conducted. Also, the focus of this research was to characterise the process features commonly used across countries in appraisal for RDTs. It does not extend to the examination of methodological approaches to better deal with RDTs. Another challenge was that despite repeated efforts to contact different experts, for five out of 37 it was not possible to create a robust vignette. However, based on public knowledge these countries do not include supplemental processes. Furthermore, the less formal aspects of the appraisal processes that are implicit or not documented may not have been captured. For example, some countries stated they do not allow for flexibility in evidence quality, but in practice they may do so. A number of other special processes/criteria that may be applied to RDTs were also identified (e.g. process for highly specialised medicines in Czech Republic), but these have not been identified systematically. Processes are also evolving rapidly; since the creation of the vignettes there have already been some updates (which are captured in this paper), but these may not capture the most recent changes. An update of these vignettes at the end of 2020 to capture any recent developments is planned. Issues related to implementation of stated appraisal processes vs. actual appraisal processes are studied in another part of our research and will be reported in the future.

## Conclusions

This comprehensive overview developed with the support of country experts has explored the use of supplemental HTA appraisal processes for RDTs in Europe, Canada and New Zealand. A wide variety of approaches have been identified that permit leniency in standard HTA processes, or in a few cases bespoke processes that involve rare disease experts. The supplemental approaches used build on the ethos of the HTA process within each particular country and may require different evidence submissions, additional assessment inputs, broader considerations in appraisal or special considerations for pricing and reimbursement. The processes enable flexibility with standard processes but seek to promote some consistency when handling RDTs within a country and are being adapted as a result of legislative direction or stakeholder feedback. It is important that as more RDTs come to market, there is transparency in national appraisal processes not just to ensure fair and accountable decisions within countries, but to determine if a common appraisal framework could be developed for RDTs or ultra-RDTs across Europe.

## Data Availability

The datasets generated and analysed during the current study are available on the IMPACT-HTA website: https://www.impact-hta.eu/country-vignettes

## Abbreviations

ACT: Anatomical therapeutic chemical classification
EEA: European Economic Area
EMA: European Medicines Agency
G-BA: Joint Federal Committee
HST: Highly Specialised Technology
HTA: Health Technology Assessment
ICER: Incremental Cost-effectiveness Ratio
IQWIG: Institute for Quality and Efficiency in Health Care
MS: Member States
NICE: National Institute for Health and Care Excellence
OMP: Orphan Medicinal Product
PACE: Patient and Clinical Engagement
QALY: Quality Adjusted Life Years
RCT: Randomised Controlled Trial
RDT: Rare Disease Treatment
SMC: Scottish Medicines Consortium
TLV: Dental and Pharmaceutical Benefits Board
ultra-RDT: Ultra-rare Disease Treatment
WTP: Willingness-to-pay
